# Lichen planus with dysphagia: an interdisciplinary, monocentric study of quality of life and depression

**DOI:** 10.3389/fmed.2025.1655291

**Published:** 2025-11-14

**Authors:** Rebecca Diehl, Annegrit Decker, Annette Schmitt-Graeff, Wolfgang Kreisel, Franziska Schauer

**Affiliations:** 1 Department of Dermatology, Medical Center-University of Freiburg, Faculty of Medicine, University of Freiburg, Freiburg, Germany; 2 Department of Medicine II, Gastroenterology, Hepatology, Endocrinology, and Infectious Diseases, Medical Center-University of Freiburg, Faculty of Medicine, University of Freiburg, Freiburg, Germany; 3 Medical Center-University of Freiburg, Faculty of Medicine, University of Freiburg, Freiburg, Germany

**Keywords:** esophageal lichen planus, quality of life, dysphagia, depression, anxiety, DLQI, disease burden

## Abstract

**Background:**

Lichen planus (LP) is an inflammatory condition affecting skin and mucous membranes. Esophageal LP (ELP) is an underrecognized form causing dysphagia, with significant potential impact on patient quality of life.

**Objective:**

To comprehensively assess quality of life, health satisfaction, and psychological burden in LP patients with dysphagia, comparing outcomes between patients with confirmed ELP versus those with dysphagia attributed to oral LP (OLP) manifestations.

**Methods:**

Prospective cohort study conducted at the University of Freiburg Medical Center including 47 patients with LP presenting with dysphagia. Following comprehensive dermatological assessment and esophagogastroduodenoscopy with biopsy, patients were categorized into ELP (*n* = 21, 45%) or non-ELP groups (*n* = 26, 55%). Patients completed validated questionnaires including the Dermatology Life Quality Index (DLQI), General Health Questionnaire-12 (GHQ-12), Patient Health Questionnaire-9 (PHQ-9), and comprehensive assessments of health satisfaction, quality of life, and symptom burden.

**Results:**

Nearly half of all patients (47%) expressed health dissatisfaction, with ELP patients showing significantly worse health satisfaction compared to non-ELP patients (*p* < 0.05). The psychological burden was substantial: 89% of patients exhibited pathological PHQ-9 scores indicating depression (42% moderate, 39% mild, 8% severe), while 55% screened positive for potential psychopathology on GHQ-12. Younger patients and women consistently reported higher disease burden across multiple measures. The mean DLQI was 7.56, with skin LP manifestations showing the highest impact (mean 9.61, *p* = 0.037). Notably, DLQI failed to capture ELP-specific burden, showing no significant difference between ELP and non-ELP groups.

**Conclusion:**

LP patients with dysphagia experience profound quality of life impairment and psychological distress, with nearly 9 in 10 patients showing signs of depression. ELP patients demonstrate significantly worse health satisfaction than non-ELP patients, yet current quality of life instruments inadequately assess ELP-specific burden. The alarming prevalence of psychological comorbidities, particularly among younger patients, necessitates routine mental health screening and integrated psychological support in LP management. These findings provide critical evidence supporting comprehensive, interdisciplinary treatment approaches and justify advanced therapeutic interventions for this challenging patient population.

## 1 Introduction

Lichen planus (LP) is an inflammatory condition affecting skin, mucous membranes and skin appendages (hair, nail). Oral LP (OLP) is the most common mucosal LP (MLP) subtype (1, 2). OLP is classified by clinical type (reticular, papular, plaque-like, atrophic, ulcerative, and bullous) and anatomical location (buccal mucosa, gingiva, tongue) (3). Multiple forms can coexist, and these variants produce varying symptoms depending on their presentation and site. For instance, the reticular pattern with Wickham’s striae is typically asymptomatic, while ulcerative or bullous forms can severely impair quality of life. Patients with these severe forms may experience intense pain when consuming spicy, acidic, or hot foods and beverages. Additionally, severe OLP can cause significant psychological distress, including negative self-image, relationship difficulties, and depression (4–8).

Esophageal lichen planus (ELP) is an underrecognized mucosal manifestation that can cause dysphagia, regurgitation, and food-related discomfort, though it may also be asymptomatic (9–12). Whereas the overall prevalence of ELP seems to be low, ELP can be found quite frequently in LP patients presenting with distinct esophageal symptoms (13). Diagnosing ELP remains challenging. We recently published simplified diagnostic criteria and a clinical questionnaire to facilitate the clinical screening of ELP patients and ensure their prompt referral for gastroscopy (13). This data were obtained from a prospective cohort in which we screened for symptomatic ELP.

The varied presentations of LP necessitate individualized approaches to clinical management, patient education, and psychosocial support (14–16). LP significantly impacts patients’ quality of life, with higher rates of depression and anxiety reported. The effects vary widely between individuals, spanning cosmetic, social, psychological, and daily life domains (4, 17–21). Previous quality of life studies focused primarily on genital and oral LP. For ELP patients large data is missing. Recent studies include only 4 ELP patients among 72 total LP cases examined (22). This has two main reasons: first, ELP is a rare manifestation, and second, ELP is potentially underdiagnosed (10, 12, 23). However, ELP affects quality of life differently than other LP manifestations. Experiencing dysphagia, which may indicate ELP involvement, can pose significant challenges to daily activities and psychological well-being. For example, patients might avoid foods that exacerbate dysphagia symptoms or those difficult to swallow, such as bread and other easily obstructive foods. As one can imagine, these dietary restrictions can significantly impact the quality of life and daily functioning of individuals suffering from ELP. Studying quality of life is necessary to demonstrate how ELP patients are impaired in their daily lives. This is especially important because ELP is a difficult-to-treat LP manifestation, making it necessary to use off-label medications when conventional treatments remain ineffective (22, 24). Given that, documenting quality of life impairment is essential for clinical decision-making and treatment justification.

In the previously described cohort (13), we therefore assessed quality of life. The cohort included patients with lichen planus (LP) who presented with dysphagia. This cohort was originally created to screen for symptomatic ELP in LP patients with dysphagia (13). We now study their quality of life regardless of whether ELP is present (first approach). Due to our study design, we have a high proportion of ELP patients. Since this is a very rare and insufficiently described LP manifestation in terms of quality of life, our second approach aims to investigate whether ELP represents a more clinically limiting condition with worse quality of life compared to the rest of the dysphagia group. By quantifying the disease burden in this underrepresented population, this study provides treating physicians concrete evidence to guide treatment decisions and demonstrates why effective therapy should be prioritized for patients with this challenging LP manifestation.

## 2 Materials and methods

Between January 2020 and December 2023, our research team carried out a prospective cohort study at the University of Freiburg Medical Center, involving both the Dermatology and Gastroenterology departments. The study population comprised two groups:

1. Patients with either an established or new diagnosis of LP who came to our clinic reporting any type of esophageal symptoms.
2. Individuals referred for endoscopic examination to investigate undiagnosed esophageal issues that were potentially indicative of ELP.

Initially we screened 77 patients in this bidirectional recruitment approach. After exclusion 47 patients underwent further investigation. ELP diagnosis was confirmed by recently published criteria (13). The treating physician completed a questionnaire regarding the clinical manifestation of LP. This considered the localization (f.e. oral, genital, skin, nail) and clinical manifestation (f.e. erosions, Wickham’s reticular lesions). The study groups were compared regarding their clinical manifestations, for example patients suffering from ELP (ELP group) versus those who did not have ELP (non-ELP group).

The Freiburg Ethics Committee granted approval for this study, assigning it the identification number 20-1227-1. The study was registered in the German Clinical Trials Register (Deutsches Register Klinischer Studien, DRKS) with the registration number: DRKS00023700. To ensure consistency in data collection, all participants completed the designated questionnaires during their initial visit to the clinic.

Personal data were extracted from the medical records. Skin condition-associated quality of life relied upon administration of the Dermatology Life Quality Index (DLQI) (25). Screening for mental wellbeing and common psychiatric pathology utilized the 12-item General Health Questionnaire (GHQ-12) (26). The GHQ12 consists of 6 positively and 6 negatively coded questions about various areas of psychopathological symptoms and is primarily used for low-threshold detection of psychological distress (27). The Linkert scoring was used to evaluate the GHQ-12 score in a one dimensional way. A score ≥11 was set as an indicator for relevant psychological distress (28). The Patient Health Questionnaire (PHQ-9) additionally evaluated degrees of depression (29). It is a brief screener for anxiety and depressive symptoms, demonstrating good internal consistency and construct validity in past studies (29). Scores from 0–9 were considered normal, 10–14 points were considered an indicator for mild depression, 15–19 points for moderate depression and scores over 20 for severe depression (29). Participants also indicated specific domains affected by LP using a checklist that included practical, familial, emotional, spiritual, and physical subsections. This checklist was designed for cancer patients to evaluate their need for help in various life domains, and we used the validated German version (30). Health satisfaction and life quality were assessed via a Likert scale ranging from 1 = very poor to 5 = very good.

The *t*-test was used to compare numeric and binary variables. The Mann-Whitey *U* test was used to compare Linkert-type-scale-numeric-values and binary variables. The Fisher’s exact test was employed to compare binary and grouped variables. Pearson’s correlation coefficient was utilized to analyze the relationship between numeric variables. *p*-values <0.5 were considered statistically significant.

The study utilized the REDCap web platform through University Hospital Freiburg to securely gather participant health data. This validated bioinformatics system allowed standardized gathering, monitoring, coordination, and examination of variables of interest across the LP cohort. REDCap’s customized infrastructure for protected research data management optimized the institutional evaluation of patients under controlled conditions (31, 32). Afterwards, the data was anonymized and analyzed using the R-Studio Software No. 2024.04.1+748.

## 3 Results

**Figure 1:**
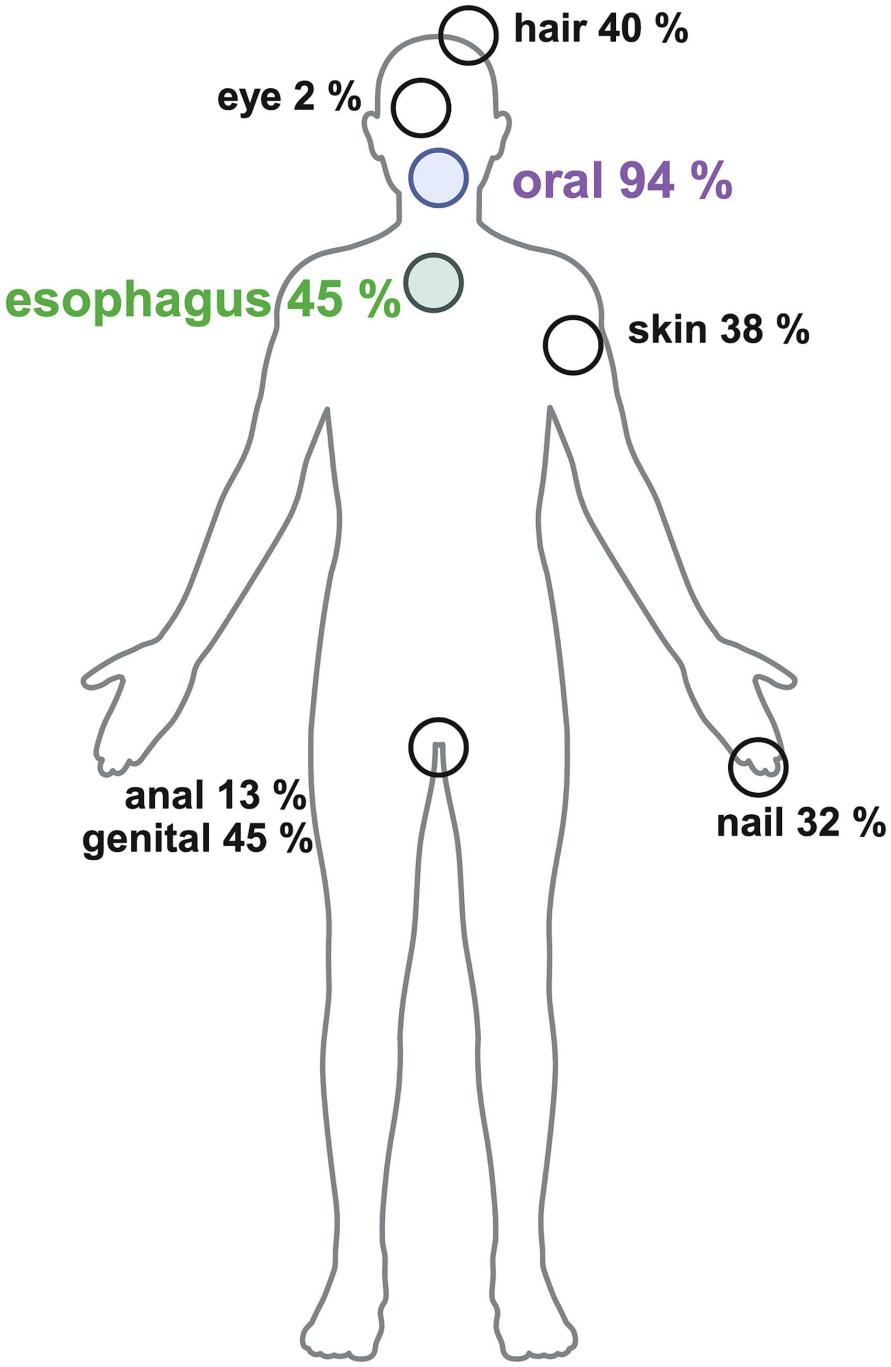
Clinical LP manifestation within the whole dysphagia cohort.

After exclusion, our analysis focused on 47 patients with dysphagia and confirmed oral and/or esophageal LP diagnosis, based on comprehensive dermatologic and endoscopic assessments. To look in more detail in the clinical LP manifestations within the whole study group please see Figure 1.

**Figure 2:**
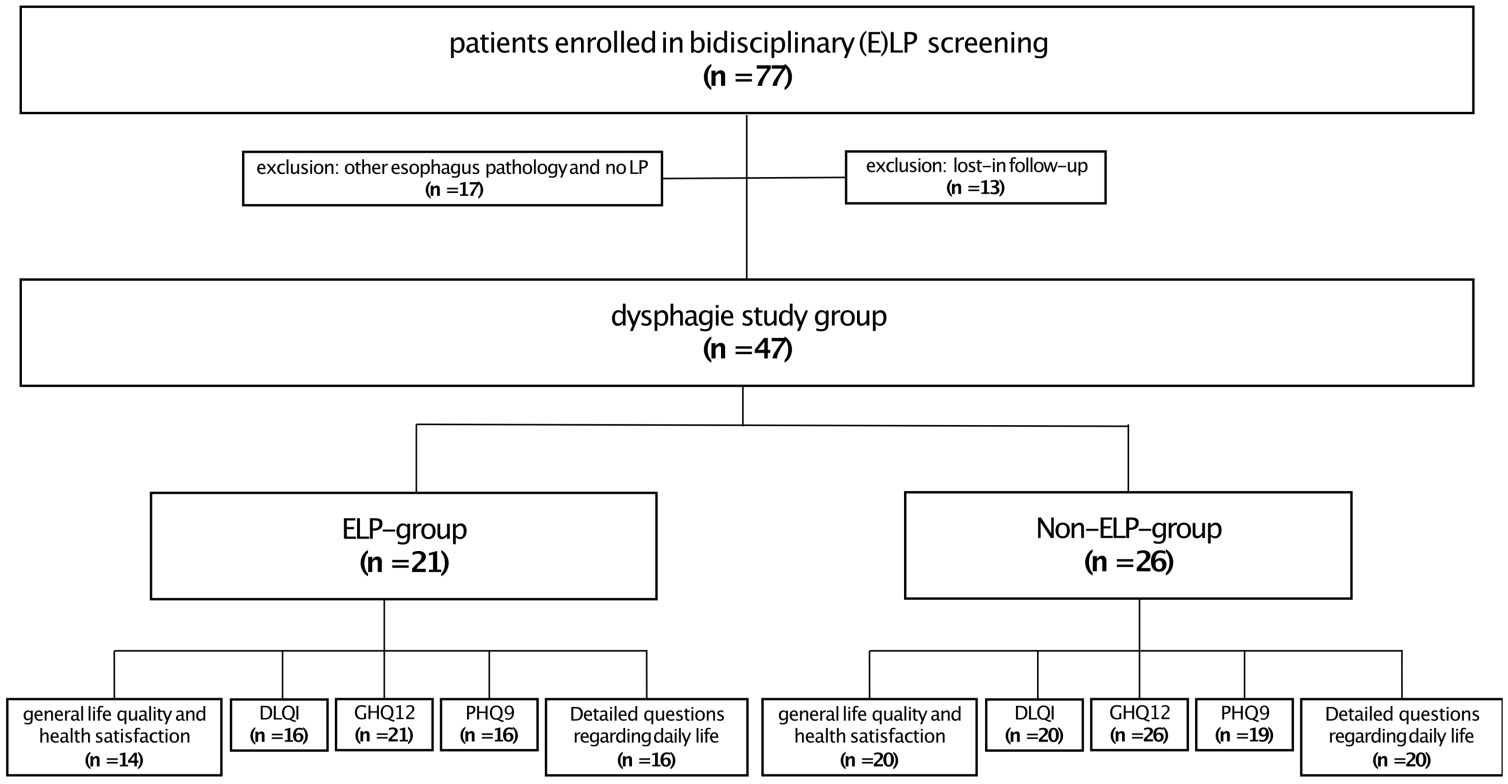
Study cohort recruitment.

Twenty-one patients (45%) were diagnosed with ELP (ELP group), while 26 patients (55%) experienced dysphagia without confirmed esophageal pathology. These patients mainly had oropharyngeal dysphagia, as 96% (25/26) of them had oral LP manifestations as the connecting LP presentation (non-ELP-group), and no other explanatory factor was identified on gastroendoscopy. It should be mentioned, that within the ELP group 90% (19/21) of the patients also had an oral involvement. On closer examination, there was no significant difference in oral involvement between the ELP and non-ELP groups. Both showed similar patterns of regional involvement (buccal mucosa, gingiva, and tongue) and comparable clinical manifestations, including Wickham’s striae and erosions/ulcerations. Regarding the questionnaires, 34 patients completed the question about general life quality and health satisfaction (*n* = 14 ELP, *n* = 20 non-ELP), 36 patients the DLQI (*n* = 16 ELP, *n* = 20 non-ELP), 47 the GHQ12 (*n* = 21 ELP, *n* = 26 non-ELP), 25 patients the PHQ9 (*n* = 16 ELP, *n* = 19 non-ELP) and 36 the detailed questions about practical, family and physical problems (*n* = 16 ELP, *n* = 20 non-ELP) (Figure 2).

### 3.1 Health satisfaction and life quality

**Figure 3:**
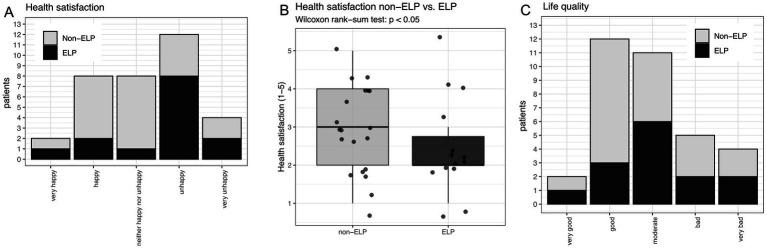
**(A)** Overall health satisfaction comparing non-ELP versus ELP patients. **(B)** Reduced health satisfaction in ELP compared to non-ELP patients (*p* < 0.05). **(C)** Overall quality of life grouped by non-ELP versus ELP (no significant difference).

Nearly half of all patients expressed dissatisfaction with their health: 35% (12/34) reported being “unhappy” and an additional 12% (4/34) reported being “very unhappy” with their health status (Figure 3A). A significant difference in health satisfaction was observed between the ELP and non-ELP groups (*p* < 0.05; Figures 3A,B), as well as in patients with anal LP manifestation (*p* < 0.05). In contrast, overall life quality was not perceived as poorly as health satisfaction. Most patients reporting either good (35%, 12/34) or moderate (32%, 11/34) life quality (Figure 3C). Only 15% (5/34) reported bad life quality, and 12% (4/34) reported very bad life quality (Figure 3C). There was no significant difference in life quality depending on the different LP manifestations or sum of LP manifestations.

### 3.2 DLQI

**Figure 4:**
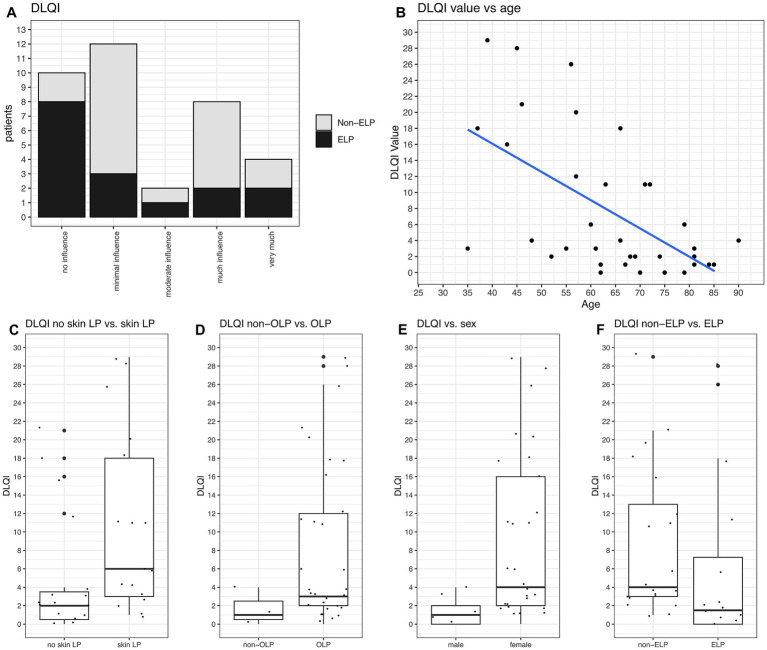
DLQI values and their correlations. **(A)** DLQI values categorized from “no influence” to “very much influence, on quality of life stratified by non-ELP and ELP groups. No significant difference is observed between the groups. **(B)** DLQI significantly correlates with age, with younger patients reporting higher burden (*p* = 0.0002). **(C)** Patients with skin manifestations had the highest DLQI values (mean 9.61; Q1: 2.50, Q3: 14.50; *p* = 0.0037). **(D)** OLP patients had significantly higher DLQI values (mean 7.63; Q1: 2.00, Q3: 11.35; *p* = 0.006). **(E)** Women reported higher DLQI values than men (mean 8.12; Q1: 2.00, Q3: 11.75; *p* = 0.0001). **(F)** No significant difference in DLQI values between non-ELP and ELP groups.

The mean DLQI was 7.56 (Q1: 1.00; Q3: 11.25) for all 36 patients filling up this questionnaire. The overall DLQI scores can be grouped into two categories: one group with no or minimal influence (22/36 patients), and another group with much or very much influence (12/36 patients). Only 2/36 patients had an intermediate value indicating moderate influence (Figure 4A).

DLQI values were significantly related to age, with younger patients reporting higher DLQI values (*p* = 0.0002, Figure 4B). There were also significant correlations between DLQI values and the type of LP manifestations. Patients suffering from skin LP had the highest DLQI values with 9.61 (Q1: 2.50; Q3: 14.50) (*p* = 0.037, Figure 4C). Moreover, patients with OLP had significant higher values than those without (mean DLQI 7.63, *p* = 0.006, Figure 4D), and women reported higher values than men (mean DLQI 9,01, *p* = 0.0001, Figure 4E). There was no difference in DLQI values regarding ELP (Figures 4A,F), genital LP, anal LP, nail LP or sum of LP manifestations.

### 3.3 Emotional and physical problems

**Figure 5:**
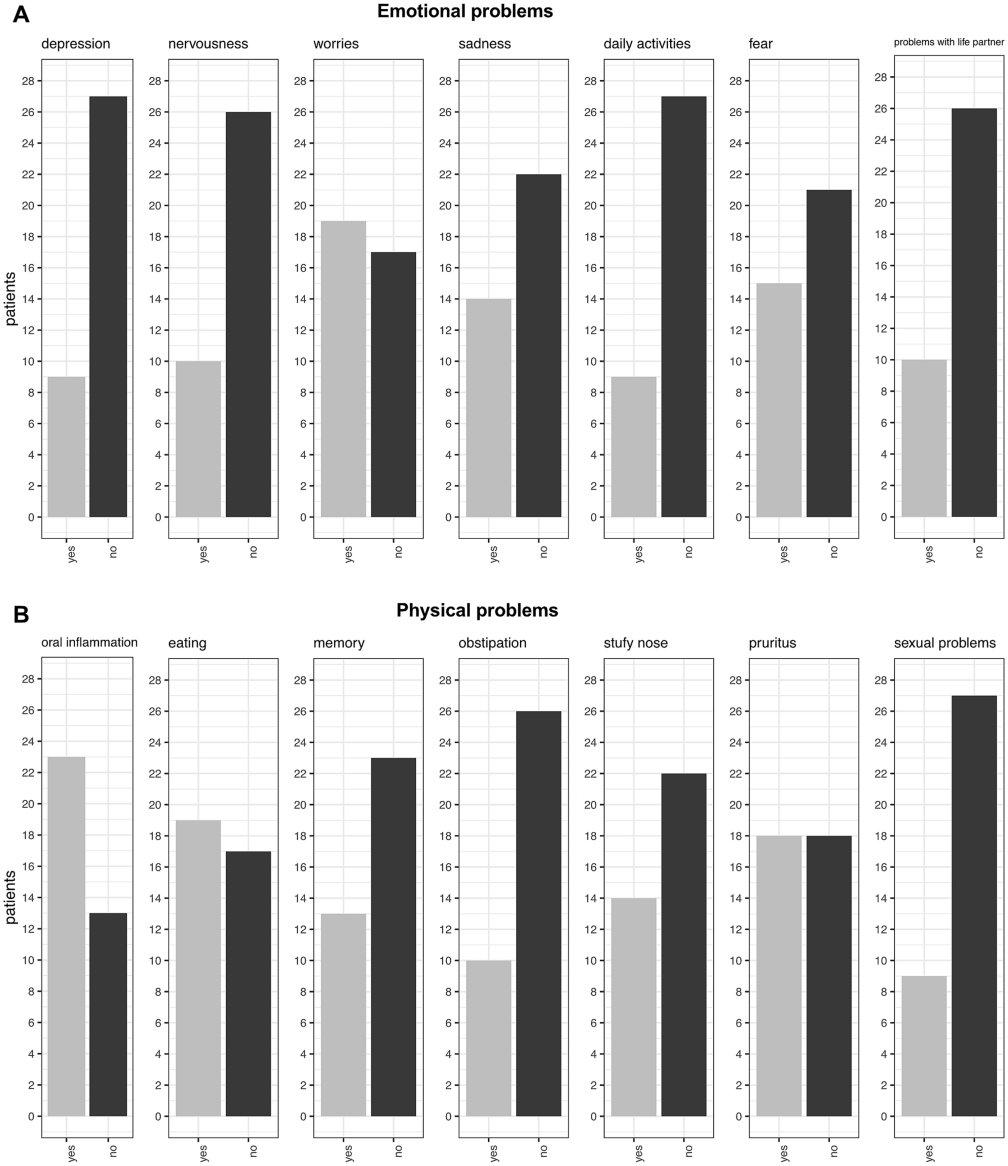
**(A)** Emotional problems. **(B)** Physical problems which were reported during the examination.

When asked about emotional problems, the most common issues reported were worries (53%, 19/36), fear (42%, 15/36), and sadness (39%, 14/36). Less common problems included issues with life partners (28%, 10/36), nervousness (28%, 10/36), and loss of interest in daily activities (25%, 9/36). Notably, when directly asked about depression, only 25% (9/36) of patients confirmed experiencing it, which contrasts with the PHQ-9 results (discussed below) (Figure 5A).

Regarding physical problems, oral inflammation was reported as the most common issue, affecting 64% (23/36) of patients, with 22 of these 23 patients having OLP as the underlying cause of their symptoms. The second most common problem was related to eating, with 53% (19/36) of patients experiencing difficulties. Pruritus ranked as the third most common issue, affecting exactly half of the patients. Memory problems were reported by 36% (13/36) of patients, while 39% (14/36) experienced nasal congestion. Constipation affected 28% (10/36) of patients, and 25% (9/36) reported sexual problems (Figure 5B).

### 3.4 GHQ12

**Figure 6:**
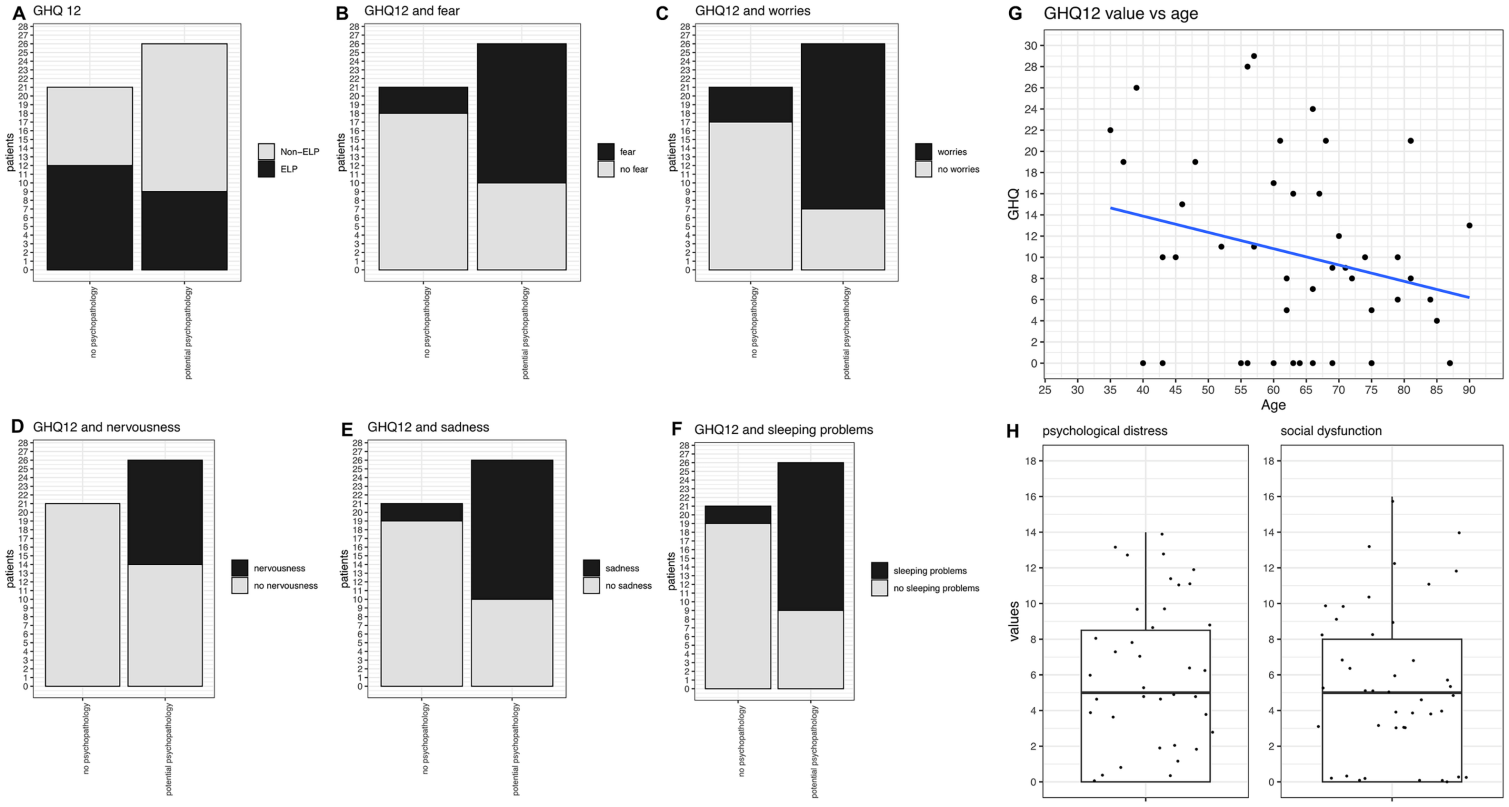
GHQ-12 analysis and correlations. **(A)** GHQ-12 values comparing ELP vs. Non-ELP groups: no significant difference observed. Twenty-six patients screened positive for potential psychopathology, while 21 did not. **(B)** Frequency of fear significantly correlates with GHQ-12 scores (*p* = 0.0012). **(C)** Frequency of worries significantly correlates with GHQ-12 scores (*p* = 0.0004). **(D)** Frequency of nervousness significantly correlates with GHQ-12 scores (*p* = 0.0004). **(E)** Frequency of sadness significantly correlates with GHQ-12 scores (*p* = 0.0003). **(F)** Frequency of sleeping problems significantly correlates with GHQ-12 scores (*p* = 0.0003). **(G)** Trend towards higher GHQ-12 values in younger patients, though not statistically significant. **(H)** Psychological distress and social distress components of GHQ-12 show similar values with no significant difference between the two components.

We used the GHQ-12 score to screen for potential psychopathology. Overall, 55% (26/47) of our study group screened positive for potential psychopathology (Figure 6A). When comparing the psychological distress and social distress components of the GHQ-12 questions, we found no difference in our group. Patients suffered equally from both distress factors (Figure 6H). There were no significant differences between GHQ12 values regarding the type of LP manifestation (as exemplified by the comparison between ELP vs. non-ELP groups, Figure 6A).

When correlating GHQ12 values with the aforementioned symptoms, patients with GHQ-12 scores indicating potential psychopathology reported significantly more frequent experiences of fear (*p* = 0.0012, Figure 6B), worries (*p* = 0.0004, Figure 6C), nervousness (*p* = 0.0004, Figure 6D), sadness (*p* = 0.0003, Figure 6E), and sleeping problems (*p* = 0.0001, Figure 6F). Moreover, they more often suffered from loss of enjoyment in daily activities (*p* = 0.0025) and memory issues (*p* = 0.0018). Regarding physical problems, they more frequently reported oral inflammation (*p* = 0.03) and nasal congestion (*p* = 0.036). The correlation between GHQ-12 values and age was not statistically significant, but there was a trend towards higher values in younger patients (Figure 6G).

### 3.5 PHQ9

**Figure 7:**
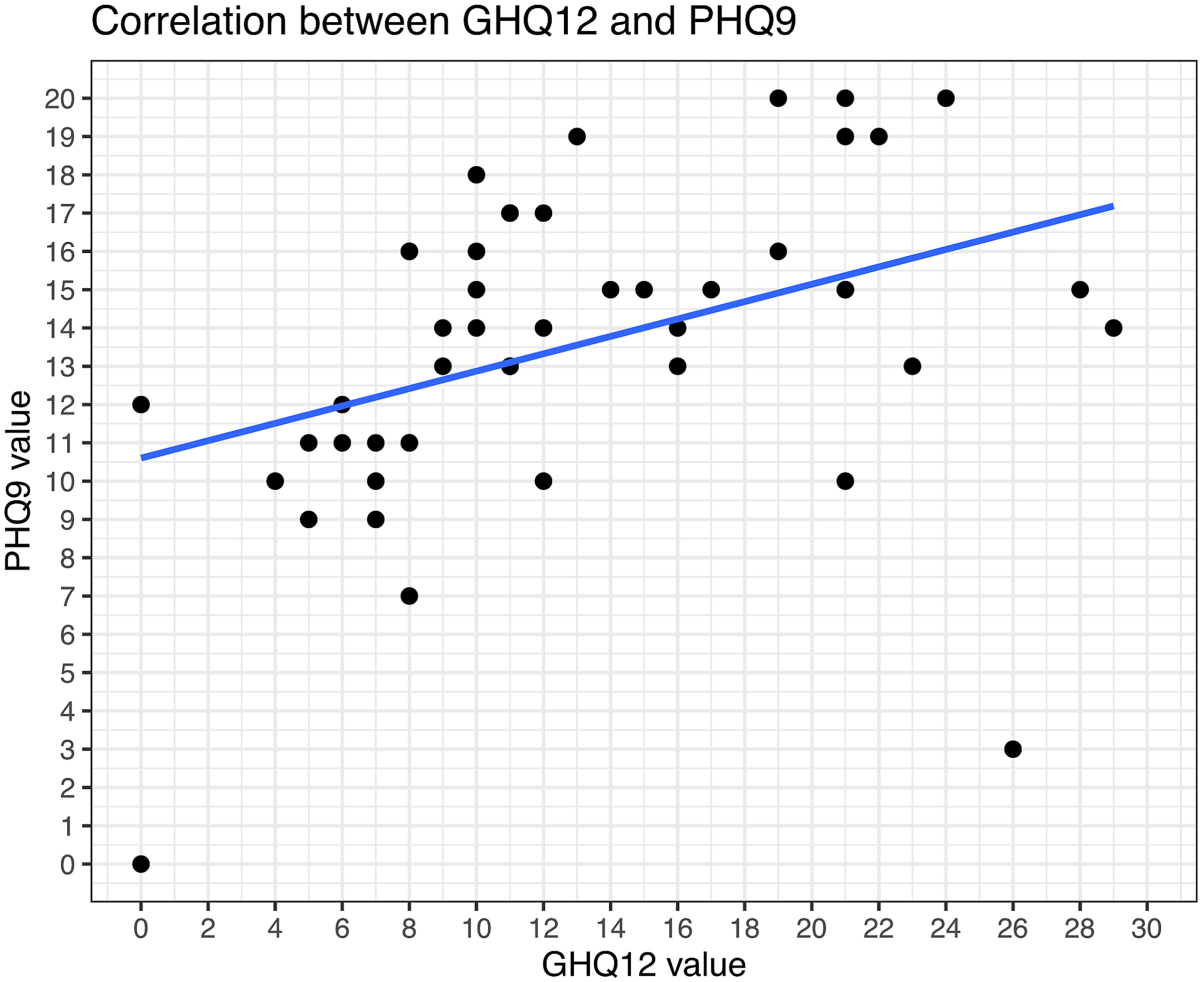
Correlation between GHQ12 and PHQ9 values.

**Figure 8:**
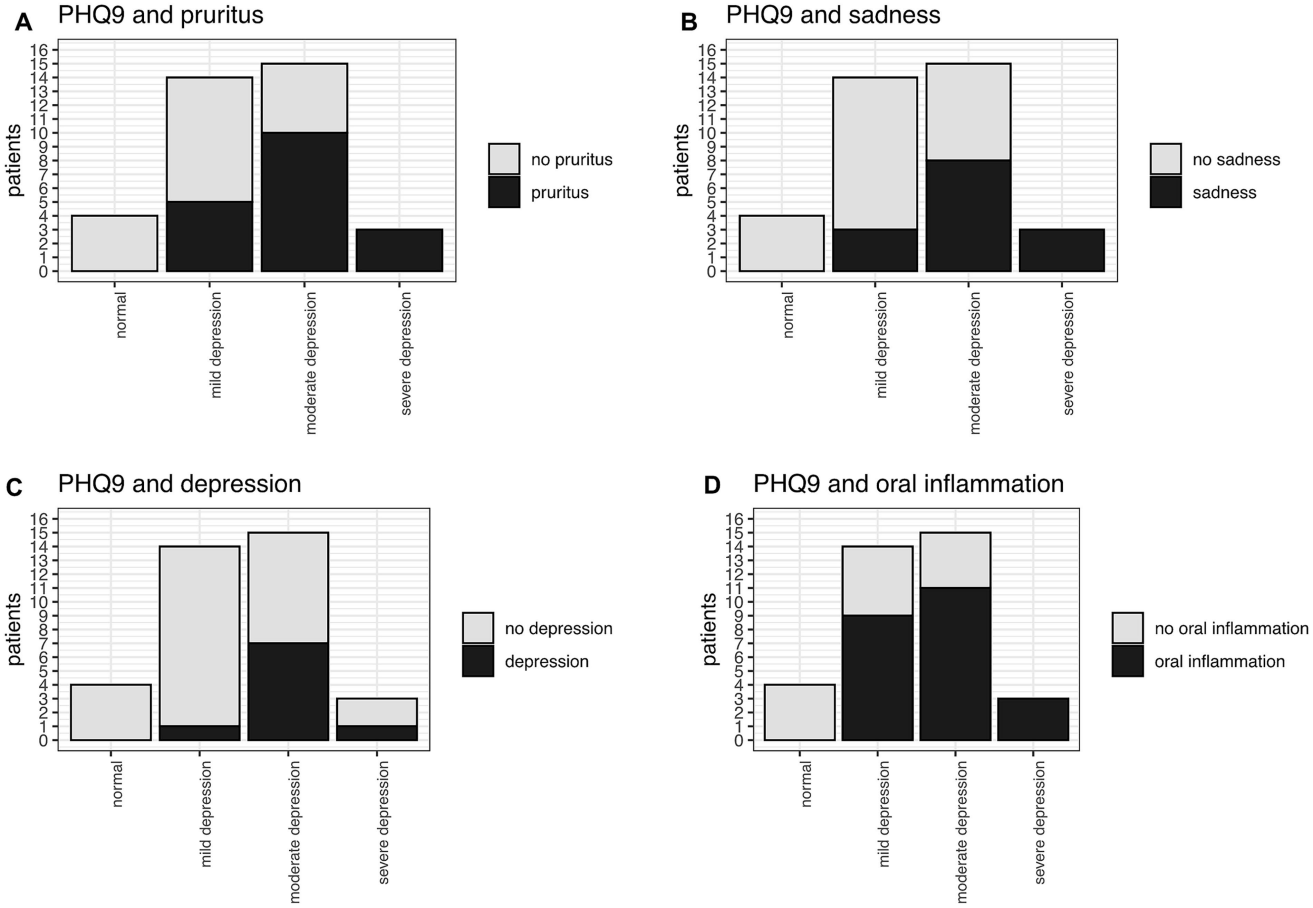
PHQ9 and significant correlation with **(A)** pruritus (*p* = 0.016), **(B)** sadness (*p* = 0.0009), **(C)** depression (*p* = 0.0049), **(D)** oral inflammation (*p* = 0.0120).

The PHQ9 and GHQ12 values intercorrelated good in our study (Figure 7). In total, 89% of the patients had PHQ-9 scores suggestive of some level of depression. 42% (15/36) were screened for moderate potential depression, while even 8% (3/36) were screened for severe depression. Mild depression was indicated in 39% (14/36) of patients (Figure 8). Higher PHQ-9 values significantly correlated with patients more frequently reporting pruritus (*p* = 0.016, Figure 8A), sadness (*p* = 0.0009, Figure 8B), depression when directly asked about it (*p* = 0.0049, Figure 8C), and oral inflammation (*p* = 0.0120, Figure 8D). There was no significant correlation between PHQ-9 scores and the type of LP manifestations, f.e. ELP vs. non-ELP or the sum of LP manifestations.

## 4 Discussion

In our quality of life study on LP, participants were preselected based on the symptom of dysphagia, which may indicate a potential esophageal involvement in patients with LP (10). All patients underwent dermatological assessment as well as esophagogastroduodenoscopy with biopsy to confirm potential ELP diagnoses. We identified symptomatic ELP in 21 of the 47 patients (13). Studying quality of life from multiple perspectives in this preselected LP group is particularly valuable because ELP patients are significantly underrepresented in LP quality of life studies. Our findings demonstrate a substantially impairment in patients’ quality of life, underscoring the urgent need for effective therapy and their prioritization across multiple levels of healthcare.

A notable finding in our study was the stark contrast between health satisfaction and overall quality of life ratings. Nearly half of all patients (47%) expressed dissatisfaction with their health, with ELP patients showing significantly worse health satisfaction compared to non-ELP patients (*p* < 0.05). However, overall quality of life assessments were more favorable, with 67% of patients reporting good or moderate quality of life. This discrepancy suggests that patients may compartmentalize their disease-specific health concerns separately from their broader life satisfaction, highlighting the importance of assessing both domains in clinical practice.

While the DLQI was specifically implemented to measure quality of life in dermatologic patients (25), its utility varied across LP manifestations. The mean DLQI of 7.56 aligns with previous LP studies, indicating moderate impact on daily life (22). Four important demographic and clinical correlations emerged: first, younger patients demonstrated higher DLQI values compared to older patients and women had higher values then men. These findings suggest that clinicians should be particularly attentive to younger patients and women when assessing disease burden and treatment needs. Secondly, patients with skin involvement showed the highest DLQI values (mean = 9.61, Q1: 2.50; Q3: 14.50, *p* = 0.037, Figure 4C), indicating a moderate impact on daily life, consistent with previous findings (33). Thirdly, OLP patients also demonstrated significant higher DLQI values than those without oral involvement (*p* = 0.0006). Previously the Oral Health Impact Profile was typically used for OLP patients (34–37), but in this study, we chose to use DLQI as a consistent screening tool for all manifestations sites. However, this findings should be interpreted cautiously, as 94% (44/47) of our cohort had OLP, leaving only 3 (3/47) patients as controls. Last and unexpectedly, ELP patients did not show elevated DLQI scores compared to non-ELP patients. This finding likely reflects a key limitation of the DLQI, which was originally developed for visible skin conditions and does not capture the specific symptoms and challenges associated with esophageal dysphagia, such as swallowing difficulties and dietary restrictions (25).

Our study reveals an alarming prevalence of psychological distress in LP patients with dysphagia. The GHQ12 score indicated relevant psychological distress in 55% of the patients, while an even more concerning 89% exhibited pathological PHQ-9 scores suggestive of depression (42% moderate, 39% mild, 8% severe). The potential depression rate in our cohort is higher than in other LP studies (33). This could be explained by the preselected group with most patients suffering from at least two LP manifestations, indicating a high disease burden. Younger patients were more vulnerable in this regard. In the subgroup analysis there was no significant influence between ELP vs. non-ELP group or between other LP manifestations. We also did not find a gender difference, as reported before (33). Overall these findings are concerning and highlight the substantial psychological burden associated with LP, particularly among younger individuals (33, 34, 38). Recent evidence suggests that psychological stress is both a trigger for LP and a consequence of the disease, creating a vicious cycle that may perpetuate and worsen symptoms. This bidirectional relationship underscores the critical importance of addressing mental health as an integral component of LP management (22). Given the high prevalence of depression and psychological distress, we strongly recommend implementing routine mental health screening using validated instruments like the PHQ-9 and GHQ-12 in all LP patients, particularly those with multiple manifestations or younger age.

Patients with pathological psychological screening scores reported significantly more frequent emotional symptoms (fear, worry, nervousness, sadness, sleep problems) and physical symptoms (oral inflammation, memory issues, nasal congestion). This constellation of symptoms reflects the heterogeneous nature of LP and highlights the need for comprehensive, interdisciplinary care. Szymczak-Paluch et al. (39) demonstrated that progressive muscle relaxation according to Jacobson could help reduce oral pain perception in LP patients. This could serve as an accessible first-line intervention while patients await specialized psychological care, particularly when rapid psychosomatic/psychological co-treatment may be delayed (39).

The first strength of our study is a large ELP cohort for a rare disease: Our study evaluated quality of life in 21 ELP patients, which represents a substantial cohort given that ELP is a rare manifestation (10, 12). Most previous LP quality of life studies have included very few ELP patients (e.g., only 4 out of 72 patients in a recent study), making our findings particularly valuable for understanding this underrepresented population (22). The collaboration between gastroenterology and dermatology enabled comprehensive assessment of both esophageal and extraesophageal manifestations. The use of multiple validated instruments provided a comprehensive assessment of different aspects of quality of life and psychological well-being.

Despite being relatively large for ELP research, our total cohort of 47 patients remains small, and we lacked a control group of patients without LP or with other dermatological conditions. This limits the generalizability of our findings. LP is highly heterogeneous in its clinical presentation and severity. Our associations should therefore be interpreted cautiously and cannot be generalized to all LP patients or all LP manifestations. Moreover, our study population was recruited from a university hospital setting, which likely enriched for patients with more severe, treatment-resistant LP manifestations. This referral bias may limit the applicability of our findings to LP patients managed in community settings. Lastly, the cross-sectional nature of our study prevents assessment of quality of life changes over time or in response to treatment interventions.

In conclusion, our study demonstrates that LP patients with dysphagia experience substantial impairment in quality of life, with nearly 90% showing signs of depression and over half screening positive for psychological distress. While the DLQI effectively captures the burden of cutaneous and oral LP manifestations, it fails to adequately assess the specific challenges faced by ELP patients, highlighting the need for disease-specific quality of life instruments for esophageal involvement. The high prevalence of psychological comorbidities, particularly among younger patients, underscores the critical need to integrate mental health screening and support into routine LP care. These findings provide essential evidence for clinicians to justify comprehensive, interdisciplinary treatment approaches and support insurance coverage for advanced therapies in this challenging patient population.

## Data Availability

The raw data supporting the conclusions of this article will be made available by the authors, without undue reservation.
